# MivacunaLA (MyshotLA): A Community-Partnered Mobile Phone Intervention to Improve COVID-19 Vaccination Behaviors among Low-Income, Spanish-Speaking, and Immigrant Latino Parents or Caregivers

**DOI:** 10.3390/vaccines12050511

**Published:** 2024-05-08

**Authors:** Yelba M. Castellon-Lopez, Alexandra M. Klomhaus, Cruz Garcia, Denise Marquez, Hilda Avila, Hannah Gravette, Ray Lopez-Chang, Brenda Ortega, Keith C. Norris, Arleen F. Brown, Luisa Blanco

**Affiliations:** 1 Department of Biomedical Sciences, Cedars-Sinai Medical Center, Cancer Research Center for Health Equity, Samuel Oschin Comprehensive Cancer Institute, Los Angeles, CA 90048, USA; denise.marquez@cshs.org; 2 Department of Medicine Statistics Core, David Geffen School of Medicine, University of California Los Angeles, Los Angeles, CA 90095, USA; aklomhaus@mednet.ucla.edu; 3 School of Public Policy, Pepperdine University, Malibu, CA 90263, USA; cruz.garcia@pepperdine.edu (C.G.); luisa.blancoraynal@pepperdine.edu (L.B.); 4 Families in Schools, Los Angeles, CA 90017, USA; hildasusyavila@gmail.com; 5 Innovate Public Schools, Los Angeles, CA 90017, USA; hgravette@innovateschools.org; 6 GPSN, Los Angeles, CA 90015, USA; rlopezchang@gpsnla.org; 7 InnerCity Struggle, Los Angeles, CA 90023, USA; brenda@innercitystruggle.org; 8 Division of General Internal Medicine and Health Services Research, Department of Medicine, David Geffen School of Medicine, University of California, Los Angeles, Los Angeles, CA 90095, USA; kcnorris@mednet.ucla.edu (K.C.N.); abrown@mednet.ucla.edu (A.F.B.); 9 Olive View-UCLA Medical Center, Sylmar, CA 91342, USA

**Keywords:** COVID-19, coronavirus vaccination, vaccine confidence, minority health, community health, mobile-based approach

## Abstract

We developed and tested MivacunaLA/MyshotLA, a community-informed mobile phone intervention, to increase COVID-19 vaccination among Latino parents/caretakers of minors in under-resourced areas of Los Angeles by addressing misinformation and building trust. We recruited Latino parents/caregivers with at least one unvaccinated child in East and South Los Angeles in the summer of 2021 and evaluated MivacunaLA as a randomized controlled trial with a wait-list control group. A difference-in-difference analysis showed Latino parents/caregivers that participated in MivacunaLA (*n* = 246), in comparison to the control group, were 15 percentage points more likely (*p* = 0.04) to report vaccination of minors aged 12–17 years, and 12 percentage points more likely (*p* = 0.03) to report a positive intention to vaccinate minors aged 2–11 years (when COVID-19 vaccines became available). Mobile phone-delivered digital interventions using videos and culturally tailored educational material to promote COVID-19 vaccine confidence can be an effective way to combat misinformation and deliver timely information to marginalized communities. Community-based participatory research approaches are crucial to advance health equity among minority communities, especially immigrant Spanish-speaking underserved communities.

## 1. Introduction

The COVID-19 pandemic magnified the need to disseminate timely COVID-19 vaccine-related information to highly impacted communities. Community-partnered research can be a particularly powerful tool to build community trust, counter the longstanding history of marginalization, and adopt community-informed approaches to improve health outcomes among underserved populations. Increasing COVID-19 vaccination rates is important among those communities that show higher COVID-19 infection and mortality rates.

In Los Angeles, California, Latino residents are twice as likely to be infected with and die from COVID-19 compared to their non-Latino White peers [1,2]. From the beginning of the pandemic through January 2022, following the Delta variant (B.1.617.2) wave, Latino residents in Los Angeles County exhibited the highest death rate among adults due to COVID-19 [3]. Health care issues, such as limited access to COVID-19 testing sites, employment-related exposure risk, shortage of primary care health services, and lower insurance coverage are linked to increased COVID-19 morbidity and mortality among Latinos [4,5,6,7]. Several structural and socioeconomic factors have also led to increased COVID-19 risk among Latinos, such as being more likely to face language barriers, ref. [6] to be essential workers [8], and to live in multi-generational households [9] or in close quarters with others [6] compared with people of other races.

Efforts to enhance the COVID-19 vaccination uptake among children are critical to minimizing the COVID-19–related impact on educational outcomes. A report by Los Angeles Unified School District (LAUSD), the second-largest school district in the United States, showed a participation gap in online learning that led to educational losses among high-needs students. Data from LAUSD students also showed that there was a lower rate of participation in online learning activities among middle and high school students who were Black, Hispanic, living in low-income households, and classified as English learners, in comparison to other racial and ethnic groups, income groups, and levels of English proficiency. Furthermore, children with parents who worked as essential or front-line workers or in low-paying jobs have experienced poor educational outcomes due to distance learning [10]. Thus, it is crucial to ensure that children can learn in person to address educational disparities, and increasing COVID-19 vaccination uptake among children is one strategy to not only keep children safe from contracting COVID-19 in the school setting but also to help families feel safer sending their children to in-person learning.

The vaccination rates for COVID-19 among younger Latinos under 18 years of age and those up to age 49 in under-resourced communities persistently lag behind the rates observed in other ethnic groups. Such disparities in vaccination rates have appeared across Los Angeles County school districts. For example, as of December 2021, only 60% of youth were fully vaccinated in the East District of LAUSD, which serves a low-income and predominantly Latino community, whereas 76% of youth ages 12–17 were fully vaccinated in Santa Monica/Malibu Unified School District, a more affluent, predominantly White school district [11]. Thus, through a community-partnered approach, we sought to develop a family-centered intervention to increase COVID-19 vaccinations among Spanish-speaking Latinos.

Mobile technology and text message-delivered interventions can increase vaccination rates [12,13]. We conducted a community-based, mobile phone-delivered intervention to increase COVID-19 vaccination rates among underserved Latino children in East and South Los Angeles and to improve their parents’ and caregivers’ intent to vaccinate them. We worked with trusted community organizations that provide social support services and resources to the Latino community to develop and implement a text-based intervention to address gaps in COVID-19 vaccine knowledge, misinformation, and disinformation [13,14,15,16,17]. Our intervention provided information from reliable sources and links to additional resources about the COVID-19 vaccines for children. To increase trust [18] and accommodate individuals with low health literacy, we provided videos delivered by Latino physicians [19] and promotoras (community health workers) in which they encouraged vaccination, sharing their personal testimonies [20,21,22]. We aimed to develop and test a 4-week culturally tailored intervention delivered by mobile phone to improve vaccination behaviors among Spanish-speaking Latino parents or caregivers. We hypothesized that a mobile-based approach for delivering COVID-19 vaccine information can effectively improve vaccination rates and the intent to vaccinate children among hard-to-reach immigrant Latino families in highly impacted and under-resourced communities.

## 2. Materials and Methods

### 2.1. Study Design, Recruitment and Procedures

Grounded in community-partnered research approaches designed to build community trust and improve health outcomes among highly impacted, low-income, immigrant, predominantly Spanish-speaking populations, we partnered with seven Latino-serving community organizations to form a community advisory board (CAB): Families in Schools, InnerCity Struggle, Innovate Public Schools, the Mexican American Opportunity Foundation, New Economics for Women, Eastmont Community Center, and Our Voice/Nuestra Voz. Staff and parent leaders affiliated with these organizations recruited participants for the study. For recruitment, we also hosted informational sessions with parents from the Facebook group “Our Voice/Nuestra Voz” and posted flyers on Facebook and Twitter. The study was approved by the <UCLA> Institutional Review Board (IRB protocol #21-000857). A community-informed mobile phone intervention was developed to provide culturally tailored vaccine information in an accessible digital format, paired with local COVID-19 vaccination resources, to Latino parents and caretakers of minors living in areas with low vaccine uptake.

We conducted MivacunaLA as a community-based randomized controlled trial (RCT) with a wait-list control group to ensure that all participants could benefit from the intervention. The treatment group received the intervention in month 1, and the control group received the intervention in month 2. We analyzed baseline data for the primary outcomes related to adult caregiver behaviors regarding COVID-19 vaccination for children at the 1-month point before the control group was exposed to the intervention. We hypothesized that our intervention would show an effect on COVID-19 vaccine uptake and intent to vaccinate after 4 weeks of being exposed to a culturally and linguistically tailored text message- and mobile phone-delivered educational curriculum.

Adult parent or caregivers were eligible for inclusion if they (1) self-identified as Latino/a, (2) were 18 years or older, (3) had at least one unvaccinated child of any aged 17 years or younger, and (4) had the means to receive text messages and review educational material online, such as a text-capable mobile phone with a web browser and internet access. Participants could also receive our program messages via email and complete our activities online using other electronic devices such as a desktop computer, laptop, or tablet. However, 94–96% of participants used a mobile phone to complete the initial survey and week 1 activities, highlighting the feasibility of a digital intervention delivered by mobile phone. Interested participants completed an online screening survey, either by themselves, or with help of staff and parent leaders from the community partner organizations who were trained by the study team. Eligible participants were invited by text message and email to provide informed consent and participate in the MivacunaLA program on our study platform. Only one parent or caregiver from each household was invited to participate.

Our intervention took place during the summer of 2021, when vaccines for 12- to 17-year-old children became available. We used a separate block randomization for July and August cohorts. Participants were randomized to receive either intervention at month 1 (treatment) or at month 2 (control). All participants completed a baseline survey and a follow-up survey at 1 month. The treatment group received educational material for 4 weeks, and the control group received a biweekly message telling them how many days were left until they were scheduled to start MivacunaLA at the beginning of month 2. After the treatment group completed the 4-week educational program, we sent all participants reminders twice a week for 2 weeks to complete the 1-month follow-up survey. Each participant received a USD 40 gift card via regular mail or email (based on stated preference) for participating in the program.

### 2.2. Study Intervention

Eligible participants in the program received a text message and email twice a week (Monday and Wednesday at noon) for 4 weeks. All program material was available in Spanish or English, and participants received the material in their preferred language. Each short text message (<160 characters in length) provided a link to a 2–3 min video (Monday) or short educational content of approximately 500 words (Wednesday). The educational curricula was developed with community input through two focus groups with youth and Latino parents or caregivers and feedback from a CAB. We created culturally and linguistically tailored videos with information about the COVID-19 vaccine from culturally congruent doctors and a personal testimony from a Latino/a parent who vaccinated his/her child. We also provided links to more information from reliable sources and instructions on how and where children could get the COVID-19 vaccine locally. The content of our program was distributed over four weeks, and in each week, we covered the following topics: (1) what is COVID-19 and how COVID-19 vaccines work, (2) COVID-19 vaccine myths and facts, (3) COVID-19 vaccine safety and efficacy in children, and (4) how to obtain COVID-19 vaccines in your community. Every week, we also provided information about how to get vaccines, with links to local vaccine sites, resources to address access barriers (i.e., county-sponsored free transportation services), and additional information about the vaccines from reliable sources.

Our educational material was designed based on prior literature [16,23,24,25,26] and experience working with the Latino community [19,27,28], two focus group sessions with parents and adolescents, and feedback from our CAB. To build trust and reinforce the social norm of getting vaccinated, we provided videos led by Spanish-speaking Latino health professionals [16,21,27]. We included in our program a publicly available video of a national Latino leader from the National Institutes of Health [29], and three videos created specifically for this intervention by a Latina primary care doctor, a Latina pediatrician, and a Latina promotora. All videos were created in Spanish with English subtitles. Participants were allowed to complete the online material at their own pace, and we provided a deadline for completion of the material of 6 weeks from starting the program. We called participants who had not completed study activities 1 week before the deadline. We used a secure online platform for data collection and completion of activities similar to the one used in the Understanding American Study (UAS) [30]. This trial was registered with clinicaltrials.gov (#NCT05234372).

### 2.3. Measures

Our primary outcomes were changes in (1) COVID-19 vaccination status from “no” or “unsure” to “yes” among minors 12–17 years and (2) intent to vaccinate minors 2–11 years old (COVID-19 vaccination authorization for minors ≤ 12 years old was not available in the summer of 2021). We assumed intent to vaccinate/vaccination status of multiple minors within the same age range and household to be consistent, resulting in one outcome variable per age range and household. The measures of COVID-19 vaccination status were adapted from the UAS to be specific to children [30]. COVID-19 vaccination status for minors 12–17 years and caregivers’ COVID-19 vaccination behaviors for minors 2–11 years in the household were collected among treatment and control groups, at baseline and in a follow-up survey at the end of the program (1-month survey).

### 2.4. Statistical Analysis

We conducted our primary analyses among those participants who completed both the baseline and 1-month surveys, thus focusing on understanding the effect of the intervention among those who actively participated in our study, either by completing the intervention (treatment) or just completing the 1-month survey (control). To better understand attrition in this sample, we tested for associations between demographic characteristics and loss to follow-up at month 1 (yes/no) using Chi-square tests.

We conducted a difference-in-differences (DID) analysis between treatment and control groups, pre- and post-intervention, on the vaccination status of minors 12–17 and on the vaccination intention of minors 2–11 years old, similarly restricted to those participants with both a baseline and 1-month survey. We estimated the absolute “risk” of the events, namely vaccination or intent to vaccinate, with an interaction term between the treatment group and an indicator for the 1-month survey to estimate the difference in the change in vaccination behaviors between the two groups, pre- and post-intervention. As we were interested in observing the magnitudes of pre-to-post changes, we used general estimating equations for our primary analyses. Statistical analyses were performed using SAS statistical software version 9.4 (SAS Institute, Cary, NC, USA).

We also used McNemar’s test, within both the treatment and control groups, to assess the change from pre- to post-intervention on the COVID-19 vaccination status of minors 12–17 (among respondents with at least one minor of this age in their household) and on the caregivers’ COVID-19 vaccination intention of minors 2–11 years old (among respondents with at least one minor of this age in their household).

We determined our required sample size based on an estimated effect size of 9%, derived from differences observed between Latinos and Whites in Los Angeles as per UAS data from March 2021. Our initial power calculations, targeting a statistical power of 80%, indicated that 319 participants per group (totaling 638 participants) would be necessary. However, our actual sample size was smaller than projected. Despite this, the observed effect size substantially exceeded our initial estimates.

## 3. Results

### 3.1. Participant Characteristics

**Figure 1:**
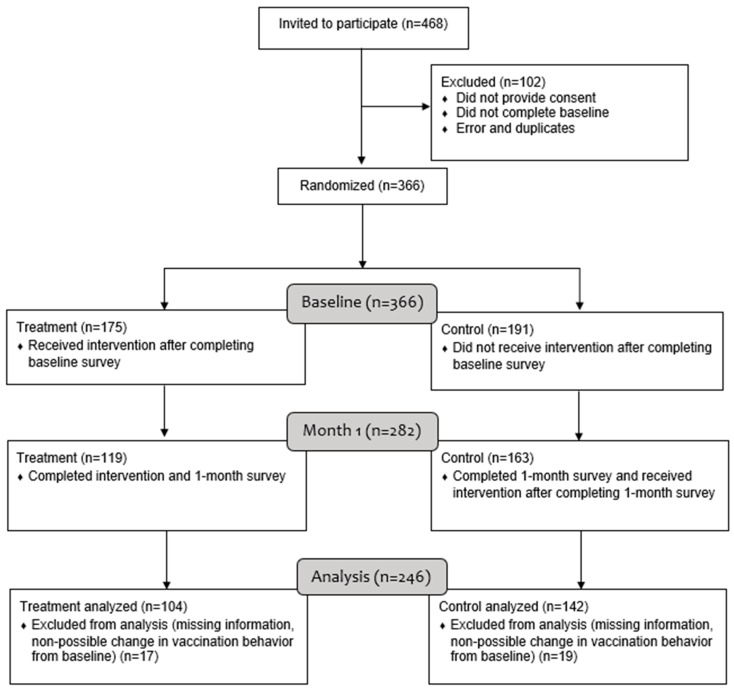
Flow diagram for data analysis.

Figure 1 presents the CONSORT Flow Diagram for our RCT. We invited 468 parents or caregivers to participate in MivacunaLA, and 366 participants completed the online informed consent and baseline survey (78% completion rate) and were randomly assigned to treatment (*n* = 175) and wait-list control (*n* = 191) groups by remote, independent, central randomization using the Mersenne Twister random number generator. Of those invited, 102 participants were excluded from the sample prior to randomization because they: (1) did not complete the consent form, (2) did not complete the baseline survey, or (3) were duplicates. Among the participants who completed the baseline survey, 119 from the treatment group and 163 from the control group also completed the month 1 survey. Our analysis excluded participants with missing data for the primary outcome and those without an unvaccinated minor in the household. Our final sample included 104 and 142 participants from the treatment and control groups, respectively.

Table 1 shows the demographics of our analytical sample. The study participants were predominantly Spanish speakers (78%) and were primarily foreign born (72%), had not completed high school (46%), and reported household incomes under USD 25,000/year (73%) and low rates of health insurance (64%). The treatment group had a higher number of foreign-born participants than the control group. A total of 85% of participants reported having children 2–11 years old in the household, and 54% reported having children 12–17 years old in the household. The treatment group had a higher number of foreign-born participants than did the control group.

Additionally, we looked for any significant differences between participants in our analysis sample who completed the 1-month survey and those who did not. We found that loss to follow-up at month 1 in our analysis sample was only associated with the group randomization (treatment versus control group, *p* < 0.01) as described above, and employment status (*p* = 0.02). We further found that those in the control group were more likely to participate in our follow-up survey, which was expected, given that control participants knew they were going to receive the educational intervention after 1 month. We are also not concerned about the differences in employment between those that completed the 1-month follow-up survey and those who did not. Nevertheless, we aggregated several employment categories status in the “other” category (Housekeeper, Retired, Disabled, Temporary Employment, Student, and “Other”). We observe that those who completed our 1-month follow-up survey were more likely to be employed.

### 3.2. Primary Outcomes

We conducted a difference-in-differences (DID) analysis between the treatment and control groups pre- and post-intervention on the vaccination status of minors 12–17 and the caregivers’ vaccination intention for minors 2–11 years old, restricted to those participants who completed the baseline and 1-month surveys. A treatment-on-treated (TOT) evaluation found no statistically significant differences between those who did and did not complete the follow-up survey. Our household-level analysis only included households if they had at least one unvaccinated child in a specific age group.

Table 2 presents the estimates from our DID analysis. We found that compared to the control group, the change in the treatment group’s “positive” intentions to vaccinate household minors 2–11 years old was 12.4 percentage points higher (*p* = 0.03), and the increase in vaccinations of household minors 12–17 years old was 15.2 percentage points higher (*p* = 0.04). Adjusting for baseline household income did not affect the interpretation of the estimates of change in vaccination behaviors (results not included due to space restrictions, but available upon request).

Results from McNemar’s tests suggest that the caregivers’ intention to vaccinate minors 2–11 years old increased significantly in the treatment group (*p* = 0.002) but not in the wait-list control group (*p* = 0.27) at 1-month follow-up. Among youth ages 12–17 years, vaccination rates increased significantly within both the treatment and the wait-list control group (*p* ≤ 0.001).

We conduct a post hoc power analysis to examine the practical significance of the magnitude of our intervention’s effect. The post hoc power analysis of the Chi-square test comparing two independent proportions using our primary analytical sample sizes of *n* = 142 in the control group and *n* = 104 in the treatment group, a baseline event rate of 0.044 in the control group and an alpha of 0.05, yielded an 88.8% power to detect a difference in proportions of 0.124, which is the effect size we found for the intention to vaccinate minors 2–11 years old. Similarly, a baseline event rate of 0.155 in the control group yielded an 80.8% power to detect a difference in proportions of 0.152, which is the effect size we found in the vaccination rates of minors 12–17 years old.

## 4. Discussion

In the evaluation of our community-based mobile phone intervention to increase knowledge about COVID-19 vaccines with an RCT design, we observed a change in behavior among Latino parents and caregivers. We find an increased likelihood of supporting COVID-19 child vaccination behaviors among those in the intervention group compared with the wait-list control group. Furthermore, compared with the controls, the participants who received the MivacunaLA intervention were 15 percentage points more likely to report vaccination of their children aged 12–17 year and 12 percentage points more likely than the controls to report a positive intention to vaccinate their 2–11-year-old children (when a COVID-19 vaccine became available). Our approach proved to be an effective way to combat misinformation and deliver timely information to low-income, Spanish-speaking, and immigrant communities who were more likely to contract COVID-19 and have a poor outcome in Los Angeles.

Through collaboration with our community partners, we created and disseminated an intervention that addressed salient themes leading to vaccination mistrust in the Latino community. Our intervention centered content around building vaccine knowledge and awareness, addressed specific myths prevalent in the Latino community, and partnered with culturally and linguistically congruent doctors who are trusted sources of information to empower families to make informed decisions.

MivacunaLA was a cross-sector, collaborative approach that utilized technology to provide participants with simple and easily accessible digital messaging via mobile phone. Text message reminders have proven to be effective at increasing immunization among adolescents [15], including influenza vaccination among pediatric Latino patients in low-income urban areas [31]. Several promising pilot studies have demonstrated that mobile texting interventions can be effectively increase human papillomavirus (HPV) vaccine uptake among underserved populations, particularly when content includes local clinic information and peer testimonials [12,13,14,15,16,17,18,19,20,21,22,23,24,25,26,27,28,29,30,31,32]. Prior literature also supports the development of culturally specific vaccine education to combat misinformation and improve vaccination behaviors [23,24,25,26,27,28,29,30,31,32,33].

Our findings support the role of mobile phone-delivered educational interventions to increase COVID-19 vaccine uptake in communities that are often considered “hard to reach”. The implementation of the text-messaging component—specifically, the distribution of a web-based curriculum with written information and videos supporting the weekly content—was associated with improved vaccine uptake and intent to vaccinate. Prior studies have recognized the ease of implementation as a benefit of mobile phone-delivered text-based interventions [17,18,19,20,21,22,23,24,25,26,27,28,29,30,31,32,33]. Moreover, mobile interventions allow organizers to incorporate new and up-to-date information in real time to address continually changing information, which was prevalent during the pandemic. Given the inevitability of new SARS-CoV-2 variants and the race to develop and disseminate new COVID-19 vaccine booster recommendations, it is important to establish trusted sources of communication with Spanish-speaking, low-income, immigrant communities in under-resourced settings. Our community-partnered randomized trial shows that MivacunaLA drove higher rates of vaccination and intent to vaccinate among Spanish-speaking, immigrant families living in under-resourced and highly-impacted communities.

Although we find these results promising, our study has some limitations. First, our analysis is based on self-reported data, with no official proof of vaccination among 12–17-year-old minors. Future interventions of this nature should aim to gather official records of vaccination status. Second, because our study took place when COVID-19 vaccines were not available for minors under 12 years old, our results are based on the perceptions of the respondents at that time, which may have changed with the availability of vaccines for this age group. Third, we designed MivacunaLA to address the informational needs of Latino families in Los Angeles; the needs and messaging may be different for different communities across the nation, which may limit the generalizability of our findings. However, we suggest that future mobile programs tailor our foundational elements to meet the needs of other communities. Fourth, we provided a USD 40 gift card for participating in our intervention. This may have created a financial incentive to complete program activities among participants that might be hard to replicate at a larger scale or in a different setting. Finally, participants’ responses may have been influenced by their desire to please the MivacunaLA team and community organizations. However, there were no incentives for positive vaccination status or responses.

## 5. Conclusions

Our study provides evidence that an educational program regarding COVID-19 vaccines and children delivered via mobile phone can be a practical and effective approach for schools, community organizations, and governments to address the informational needs of Latino families and, especially, immigrant communities. As COVID-19 vaccines are now available for children 6 months or older, it is of great importance that we implement educational programs for parents and caregivers of children in underserved communities to build confidence in COVID-19 vaccination among children. Programs such as MivacunaLA that empower minority families to make informed decisions are necessary for addressing COVID-19 disparities.

## Figures and Tables

**Table 1:** Demographic characteristics of overall sample and control and treatment groups.

Characteristic	Overall (*n* = 246)	Control (*n* = 142)	Treatment (*n* = 104)	*p*-Value ^2^
	*n* (% ^1^)	*n* (%)	*n* (%)	
Language				
English	53 (21.5)	36 (25.4)	17 (16.3)	0.09
Spanish	193 (78.5)	106 (74.6)	87 (83.7)	
Parent COVID-19 Vaccination Status				
Vaccinated	176 (71.5)	96 (67.6)	80 (76.9)	0.28
Not Vaccinated	65 (26.4)	43 (30.3)	22 (21.2)	
Unsure	5 (2.0)	3 (2.1)	2 (1.9)	
Ethnicity				
Not Hispanic/Latino/Spanish Origin	6 (2.4)	4 (2.8)	2 (1.9)	0.85
Mexican/Mexican American/Chicano	189 (76.8)	110 (77.5)	79 (76.0)	
Other Hispanic/Latino/Spanish Origin ^3^	49 (19.9)	27 (19.0)	22 (21.2)	
Missing	2 (0.8)	1 (0.7)	1 (1.0)	
Born in the U.S.				
Yes	51 (20.7)	39 (27.5)	12 (11.5)	0.01
No	176 (71.5)	92 (64.8)	84 (80.8)	
Prefer Not to Respond	19 (7.7)	11 (7.7)	8 (7.7)	
Highest Education Attained				
Some High School or Less	113 (45.9)	59 (41.5)	54 (51.9)	0.21
High School Graduate/GED	72 (29.3)	47 (33.1)	25 (24.0)	
Some College or More	61 (24.8)	36 (25.4)	25 (24.0)	
Employment Status				
Employed	89 (36.2)	57 (40.1)	32 (30.8)	0.24
Unemployed	37 (15.0)	22 (15.5)	15 (14.4)	
Other ^4^	111 (45.1)	57 (40.1)	54 (51.9)	
Do Not Know/Prefer Not to Respond	8 (3.3)	6 (4.2)	2 (1.9)	
Missing	1 (0.4)	0 (0.0)	1 (1.0)	
Household Income				
<USD 25,000	180 (73.2)	104 (73.2)	76 (73.1)	0.87
USD 25,000–USD 49,000	41 (16.7)	24 (16.9)	17 (16.3)	
>USD 50,000	23 (9.3)	12 (8.5)	11 (10.6)	
Missing	2 (0.8)	2 (1.4)	---	
Health Insurance Status				
Insured ^5^	158 (64.2)	97 (68.3)	61 (58.7)	0.23
Not Insured	56 (22.8)	27 (19.0)	29 (27.9)	
Do Not Know/Prefer Not to Respond	32 (13.0)	18 (12.7)	14 (13.5)	
Marital Status				
Currently Married	123 (50.0)	62 (43.7)	61 (58.7)	0.13
Cohabitation (Common Law Marriage)	46 (18.7)	30 (21.1)	16 (15.4)	
Widowed/Divorced/Separated	28 (11.4)	17 (12.0)	11 (10.6)	
Never Married	49 (19.9)	33 (23.2)	16 (15.4)	
Type of Household				
Married With Children	143 (58.1)	74 (52.1)	69 (66.3)	0.18
Single/Married Without Children	13 (5.3)	10 (7.0)	3 (2.9)	
Single With Children	34 (13.8)	21 (14.8)	13 (12.5)	
Other	24 (9.8)	17 (12.0)	7 (6.7)	
Do Not Know/Prefer Not to Respond	32 (13.0)	20 (14.1)	12 (11.5)	
Any Minors in Household Under 2				
Yes	32 (13.0)	15 (10.6)	17 (16.3)	0.21
No	214 (87.0)	127 (89.4)	87 (83.7)	
Any Minors in Household 2–11 Years				
Yes	209 (85.0)	121 (85.2)	88 (84.6)	0.51
No	37 (15.0)	21 (14.8)	16 (15.4)	
Any Minors in Household 12–17 Years				
Yes	133 (54.1)	71 (50.0)	62 (59.6)	0.20
No	113 (45.9)	71 (50.0)	42 (40.4)	
Other (continuous variables)				
Age of Parent (Mean, SD) ^6,7^	39.2 (8.8)	38.8 (9.5)	39.7 (7.7)	0.43
Number of Minors in Household (Mean, SD)	2.2 (1.0)	2.2 (0.8)	2.3 (1.1)	0.23

^1^ (%) reflects column percentage; in the overall column, % reflects percentage of overall participants, and in the group columns, % reflects the percentage of respondents within a group. We include here summary statistics only for the sample considered in the analysis (includes participants that completed 1-month follow-up survey and excludes participants who were already vaccinated themselves, either without a minor or with only already-vaccinated minors in the household at baseline). ^2^ We tested for significant differences between treatment and control groups and present *p*-values for Chi-square test for categorical variables and two-sample *t*-test for continuous variables; Chi-square tests exclude “missing” categories. ^3^ Includes: Puerto Rican, Cuban, Multiple Ethnicities, and “Other”. ^4^ Includes: Housekeeper, Retired, Disabled, Temporary Employment, Student, and “Other”. ^5^ Includes: government insurance, insurance through the VA, private insurance, and Medicare. ^6^ Control group: *n* = 136 parents or caregivers had a non-missing age, *n* = 137 had a non-missing number of minors; Treatment group: *n* = 100 had a non-missing age, *n* = 103 had non-missing number of minors. ^7^ Standard deviation.

**Table 2:** Unadjusted DID results assessing change from baseline to 1-month follow-up.

	Baseline	1-Month Follow-Up	Change, Δ (95% CI)	*p*-Value
Intention to Vaccinate Minors 2–11 years old				
Control	71.0%	75.4%	4.4% (−1.6%, 10.4%)	0.15
Tx	71.6%	88.4%	16.8% (7.7%, 25.8%)	<0.001
Difference			12.4% (1.5%, 23.2%)	0.03
Vaccination of Minors 12–17 years old				
Control	52.1%	67.6%	15.5% (7.1%, 23.9%)	<0.001
Tx	50.0%	80.7%	30.7% (19.2%, 42.1%)	<0.001
Difference			15.2% (0.9%, 29.4%)	0.04

## Data Availability

The data are unavailable due to privacy or ethical restrictions.
